# Perceptions of Nursing Undergraduates Regarding Their Clinical Placements: A Qualitative Interpretative Study

**DOI:** 10.3390/nursrep16090337

**Published:** 2026-09-15

**Authors:** Zaida Santana-Casiano, Claudio-Alberto Rodríguez-Suárez, Sergio Mies-Padilla, Milagros de la Rosa-Hormiga, Candelaria de la Merced Díaz-González, Héctor González-de la Torre

**Affiliations:** 1 Department of Nursing, Faculty of Healthcare Science, University of Las Palmas de Gran Canaria (ULPGC), 35016 Las Palmas de Gran Canaria, Spain; zaida.santana111@alu.ulpgc.es (Z.S.-C.); milagros.delarosa@ulpgc.es (M.d.l.R.-H.); candelaria.diazg@ulpgc.es (C.d.l.M.D.-G.); 2 Research Support Unit, Insular Maternal and Child University Hospital Complex, Canary Health Service, 35016 Las Palmas de Gran Canaria, Spain; sergio.mies@pdi.atlanticomedio.es; 3 Faculty of Healthcare Science, University of Atlántico Medio (UNAM), 35017 Las Palmas de Gran Canaria, Spain

**Keywords:** nursing students, nursing education research, nursing faculty practice, education, nursing, baccalaureate, qualitative research, educational measurement

## Abstract

**Background/Objectives**: Clinical placements are central to nursing education, but students’ experiences are shaped by the clinical learning environment, relationships with staff, autonomy, and assessment, as well as curricular design. This study explored nursing undergraduates’ lived experiences of clinical placements at the University of Las Palmas de Gran Canaria (ULPGC), their perceptions of the current placement programme, and suggestions for improvement. **Methods**: A qualitative, interpretative, sequential study with a hermeneutic phenomenological orientation was conducted. Eligible third- and fourth-year nursing students were recruited during the 2025/2026 academic year (N = 14). Phase 1 comprised ten individual semi-structured interviews, analysed inductively using ATLAS.ti, with descriptive co-occurrence patterns supporting the interpretative synthesis. Phase 2 consisted of a focus group with four additional students, with the data analysed deductively using the Phase 1 thematic framework. **Results**: Fifteen subthemes were organised into five themes: general experience, relationship with staff, autonomy and learning, structure and organisation, and assessment. Participants generally perceived their clinical placements positively, although experiences varied across settings. They associated satisfaction and learning with team inclusion, relationships with clinical staff, progressive autonomy, and supervision, while identifying challenges in placement organisation and assessment, particularly the reliance on written assignments rather than direct observation of clinical performance. Across both phases, participants’ accounts reflected interconnected relational, educational, organisational, and assessment-related dimensions. Collective discussion further elaborated these experiences and potential improvements concerning supervision, placement organisation, and assessment. **Conclusions**: Nursing students’ accounts suggested that clinical placement experiences reflected the interaction of relational, educational, organisational, and assessment-related dimensions. Collective reflection further elaborated these experiences and enabled potential improvements concerning supervision, placement organisation, and assessment to be discussed. These context-specific findings may inform future curricular review, although their transferability and the feasibility of the proposed improvements require evaluation in broader and more diverse student populations.

## 1. Introduction

Clinical placements are a cornerstone of nursing education, enabling students to apply classroom learning in real clinical settings while progressively developing their professional identity [1]. Within curricula aligned with the European Higher Education Area, the Practicum usually accounts for approximately half of the competencies that students are expected to acquire and demonstrate, highlighting its central role within the nursing curriculum [1,2]. Beyond the acquisition of techniques and procedures, clinical placements represent a process of professional socialisation through which students build confidence, self-assurance, and a sense of belonging to the profession [3,4]. In addition, previous research has shown that students’ experiences during clinical placements may significantly influence their future career plans [5,6,7,8].

However, the quality of this experience does not depend solely on curricular design but also on a broad set of contextual factors captured within the concept of the clinical learning environment (CLE) [1,9,10]. Several systematic reviews have shown that nursing students’ perceptions of their CLE may be influenced by the organisation of clinical units or services, the prevailing psychosocial climate, the quality of the supervision received, and the level of engagement of both academic and clinical staff in students’ learning [10,11]. When these conditions are unfavourable, students tend to experience insecurity, demotivation, and a reduced sense of belonging to the team [10]. These factors are associated with poorer learning outcomes and even with a higher risk of dropping out of the programme [10,12,13]. In this regard, belongingness has emerged in recent years as a central construct for understanding students’ clinical experiences, as it may mediate the relationship between the relational climate of the clinical service and students’ active involvement in care [3,4,14,15]. In the Spanish context, some studies have reported similar findings, describing how an unfavourable clinical environment—marked by staff workload or a lack of welcoming attitudes—negatively affects both learning and the emotional well-being of students during clinical placements [16,17].

Within this relational framework, clinical supervision plays a central role as a facilitator of the educational process. Evidence indicates that the quality of clinical supervision, the tutor’s specific training in mentoring competencies, and the coherence between the objectives of the training plan and daily practice in the clinical unit or service are key factors associated with nursing students’ satisfaction and learning [18,19,20]. However, evidence remains inconclusive regarding the optimal model of clinical supervision. While some studies argue that the presence of several clinical reference professionals enriches the student experience, others support the continuity of a single tutor as a means of ensuring a stronger relationship of trust [21,22]. In addition, some studies have reported incivility, rejection, or discrimination by healthcare staff towards students during clinical placements [23,24,25].

Another essential aspect to consider is the assessment of clinical competencies during clinical placements. Reflective journals, practice diaries, and portfolios are commonly used to assess clinical learning and are justified by their ability to stimulate critical thinking and strengthen the relationship between students and tutors [26,27]. However, several authors have also pointed out their limitations, including the workload they impose on students and the risk of subjectivity in grading, particularly when assessment is based more on the narrative quality of the document than on direct observation of students’ clinical performance [20,26,28,29]. The most appropriate methods and tools for assessing clinical placements in nursing education therefore remain a matter of debate.

Finally, the temporal organisation of rotations—that is, their duration and distribution across clinical services or units—is another factor that the literature has consistently associated with student satisfaction [13,30]. Available evidence suggests that longer rotations are associated with a stronger supervisory relationship and a more favourable perception of the learning environment [31], whereas excessively short rotations may limit students’ adaptation to the clinical service and increase their anxiety [32]. However, there is still no agreement on the optimal duration of rotations, and alternative models, such as longitudinal rotations, have been proposed to promote continuity of care and the consolidation of learning [33,34].

For all these reasons, further research is needed to explore and integrate the different elements that influence nursing students’ perceptions and satisfaction during clinical placements. Although qualitative studies conducted in Spanish universities have examined the clinical learning environment and students’ experiences during the COVID-19 pandemic [16,17], evidence remains limited regarding how students experience and evaluate specific clinical placement programmes within their local curricular and organisational contexts. At the ULPGC, no previous qualitative study has comprehensively examined students’ experiences of the current clinical placement programme or specifically explored their perspectives on potential improvements to the programme. According to Order CIN/2134/2008 of 3 July, issued by the Spanish Ministry of Science and Innovation (Ministerio de Ciencia e Innovación, CIN), the Practicum represents 35% of the total workload of the Nursing Degree curriculum [35]. Generating context-specific evidence from students’ perspectives may therefore provide a relevant basis for informing future reviews of the programme.

At the ULPGC, clinical placements in the Nursing Degree begin in the second year, with two two-week placements (Practicum I); continue in the third year, with four five-week placements (Practicum II and III); and culminate in the fourth year, with four two-month placements (Practicum IV and V). Altogether, the Practicum modules comprise 84 European Credit Transfer and Accumulation System (ECTS) credits, of which 30 correspond specifically to Practicum IV and 24 to Practicum V. This sequential structure, characterised by increasing autonomy and responsibility, provides the educational context examined in the present study.

Building on previous qualitative research identifying relational, supervisory, educational, and organisational factors that influence nursing students’ clinical learning experiences, the present study addresses this local knowledge gap by examining these dimensions, together with assessment-related experiences, within the specific curricular context of the ULPGC. Its sequential design extends this examination by using individual interviews to explore students’ experiences and a subsequent focus group to revisit and further elaborate these findings through collective reflection and to discuss potential improvements to the clinical placement programme. This approach seeks to provide an integrated, context-specific understanding of students’ clinical placement experiences while identifying areas for curricular improvement from the students’ own perspective.

Accordingly, this study aimed to explore nursing undergraduates’ lived experiences during clinical placements and their perceptions of the current clinical placement programme at the ULPGC. Specifically, it sought to identify the factors influencing students’ satisfaction, examine their perceptions of the structure and organisation of placements, identify perceived shortcomings, and gather suggestions for improvement.

## 2. Materials and Methods

### 2.1. Study Design

A qualitative, interpretative, sequential study with a hermeneutic phenomenological orientation was conducted [36]. Hermeneutic phenomenology provided the overarching epistemological and interpretative orientation of the study, directing attention to how nursing students experienced and made sense of clinical placements within their educational, relational, and organisational contexts. It was not used as a separate analytical method. Thematic analysis was used as the analytical procedure for identifying, organising, and interpreting patterns of meaning across participants’ accounts. Thus, hermeneutic phenomenology informed the interpretative perspective of the study, whereas thematic analysis provided a systematic procedure for engaging with and organising the experiential data. 

The sequential design comprised two interconnected phases: inductive thematic analysis in Phase 1, followed by directed deductive thematic analysis in Phase 2, using the Phase 1 thematic framework as a sensitising analytical structure to explore how the initial findings were further elaborated, nuanced, or challenged through collective discussion.

The study was reported in accordance with the Consolidated Criteria for Reporting Qualitative Research (COREQ) guidelines [37], which provide a framework for transparent and comprehensive reporting of qualitative studies.

### 2.2. Setting, Participants, and Recruitment

The ULPGC is a public university located in the Canary Islands, Spain, offering the Nursing Degree across three campuses: Gran Canaria, Fuerteventura, and Lanzarote. The study population consisted of third- and fourth-year nursing undergraduates enrolled during the 2025/2026 academic year at the Gran Canaria campus who were undertaking their clinical placements (Practicum) at the time of the study. Eligibility was purposively restricted to third- and fourth-year students because they had accumulated greater experience across multiple clinical placements than students in earlier years, enabling them to reflect on different aspects of the organisation, supervision, assessment, and overall experience of the clinical placement programme.

A total of 300 students met the eligibility criteria, and all were invited to participate through direct face-to-face contact and/or an invitation sent to their institutional email accounts by the principal researcher (Z.S.-C.); no prior expression of interest or registration was required. For Phase 1, ten students responded to the invitation, and all were interviewed. For Phase 2, 19 students expressed interest in participating in a focus group. Four were available and participated, whereas the remaining 15 were unable to attend that session. Participants took part in only one phase of the study.

Participants were informed that the principal researcher was a fourth-year nursing undergraduate conducting the study as part of her final degree project and that its purpose was to inform future revisions of the clinical placement programme. Before providing informed consent, students were informed that participation was entirely voluntary, that they could decline to participate or withdraw without academic or personal consequences, and that the information they provided would be treated confidentially and reported without personally identifying information. No student who agreed to participate subsequently withdrew from the study. No repeat interviews or focus groups were conducted.

### 2.3. Research Team and Reflexivity

The research team consisted of the principal researcher (Z.S.-C., female), a fourth-year nursing undergraduate at the ULPGC with previous training but limited experience in qualitative research, and five university lecturers who collaborated in the study. The collaborating researchers comprised three men and two women, all of whom held doctoral degrees; none had teaching, assessment, or other responsibilities related to the clinical placement programme. Among them, H.G.-d.l.T. (male) and C.-A.R.-S. (male) had extensive experience in qualitative research and were directly involved in the analytical process.

As the principal researcher was herself a nursing undergraduate who had previously completed clinical placements, she had first-hand knowledge of the academic and clinical context in which the study was conducted. This experience fostered a personal interest in the research topic, particularly in relation to students’ experiences during clinical placements and their perceptions of the curriculum.

This proximity to the phenomenon under study may have shaped the principal researcher’s preconceptions and expectations regarding participants’ experiences and, in the case of participants with whom a prior peer relationship existed, may also have influenced participants’ disclosure or contributed to socially desirable responses during the interviews. Rather than assuming that these influences could be eliminated, they were explicitly recognised as part of the interpretative process. During data collection, semi-structured interviews with open-ended questions were used to allow participants to describe their experiences in their own words, and the interviewer sought to avoid leading questions or responses that could unnecessarily direct participants’ accounts. During analysis, emerging interpretations were discussed regularly within the research team, including researchers who did not share the principal researcher’s position as a nursing student. These discussions were used to question assumptions arising from proximity to the phenomenon, consider alternative interpretations, and ensure that the final analytical framework remained grounded in participants’ accounts. Discrepancies in coding or interpretation were discussed until consensus was reached. No formal reflexive journal or systematic record of the interviewer’s personal reactions was maintained; therefore, reflexivity relied primarily on explicit acknowledgement of researcher positionality and collective critical discussion throughout the analytical process.

### 2.4. Data Collection

A two-phase data collection strategy was used. First, ten individual semi-structured interviews were conducted using a flexible interview guide (Supplementary Table S1). The interview guide was developed by the research team based on the study objectives and informed by previous literature on nursing students’ clinical placement experiences. It addressed the overall clinical placement experience, relationships with clinical staff, autonomy and learning, placement organisation, and assessment. An initial target of ten individual interviews was established as an a priori sampling decision, based on the recommendations of Hernández-Sampieri and Mendoza [38] for qualitative studies addressing a well-defined phenomenon. This initial target was used to guide recruitment and was not considered, in itself, evidence of sample adequacy, which was subsequently assessed during analysis in terms of informational sufficiency. 

The interviews were conducted by the principal researcher (Z.S.-C.), had a mean duration of approximately 14 min (range: 9–23 min), and were carried out between 3 October and 11 November 2025. Although relatively brief, the interviews focused on a clearly delimited phenomenon with which all participants had direct experience. The flexible semi-structured format allowed the interviewer to use probing questions, when appropriate, to encourage participants to elaborate on experiences and meanings relevant to the study objectives. Of the ten individual interviews, five were conducted face-to-face at the Faculty and five were conducted online via Microsoft Teams^®^. The only procedural difference concerned the recording method: face-to-face interviews were audio-recorded using the principal researcher’s smartphone (Redmi Note 12 Pro+, Xiaomi Communications Co., Ltd., Beijing, China), whereas the built-in Microsoft Teams^®^ recording function was used for online interviews. All interviews were transcribed verbatim prior to analysis. No people other than the participants and the researchers were present during the interviews or the focus group. 

After the individual interviews, a focus group was conducted with four additional participants and moderated by two members of the research team (Z.S.-C. and H.G.-d.l.T.). The session took place on 17 December 2025 in a classroom at the Faculty of Health Sciences, ULPGC, and lasted 1 h and 40 min. The focus group constituted the second phase of the sequential design and was used as a complementary source of data to further explore and refine the Phase 1 findings through interaction, collective meaning-making, and shared reflection, rather than as a validation or member-checking procedure.

The session was organised into two parts. The first aimed to revisit and further explore the themes and subthemes identified in the individual interviews, focusing on the overall experience of clinical placements, relationships with staff, autonomy, organisation, and assessment. The second focused on identifying problems and discussing potential improvements, encouraging participants to reflect critically on the issues identified during Phase 1. A separate focus group discussion guide, developed from the Phase 1 findings, was used for this purpose (Supplementary Table S2). Although the guide was structured around the Phase 1 findings, it was used flexibly, and participants were encouraged to introduce alternative perspectives, experiences, or issues not represented in the initial thematic framework. The moderators introduced additional probing questions when participants raised new or insufficiently explored issues, seeking clarification, concrete examples, further elaboration, or alternative perspectives rather than confirmation of the Phase 1 findings. Nevertheless, because the guide was explicitly informed by the Phase 1 findings, its potential to orient the discussion towards previously identified themes and subthemes cannot be excluded. The guide was reviewed by two researchers with experience in qualitative methods but was not pilot tested. The focus group was audio- and video-recorded and subsequently transcribed verbatim. Audio was recorded using the same device employed for the face-to-face individual interviews, while an iPad^®^ (Apple Inc., Cupertino, CA, USA) was used for video recording. In addition, the principal researcher (Z.S.-C.) recorded field notes documenting relevant aspects of group dynamics and non-verbal interactions.

For both the individual interviews and the focus group, participants’ demographic and academic characteristics were recorded, including age, gender, academic year, employment status, and previous educational background.

### 2.5. Data Analysis

Thematic analysis was conducted according to Braun and Clarke’s six-step approach [39] across two sequential phases. The hermeneutic phenomenological orientation informed the interpretation of participants’ accounts throughout both phases, with attention to the meanings attributed to their experiences and to the educational, relational, and organisational contexts in which those experiences occurred.

Phase 1 involved an inductive thematic analysis of the individual interviews, whereas Phase 2 used a directed deductive thematic analysis of the focus group, with the Phase 1 findings serving as the initial analytical framework.

Before analysis, all transcripts were anonymised by removing participants’ names and replacing them with coded identifiers. Individual interview participants were identified by the prefix “P” followed by their assigned number (P1–P10), whereas focus group participants were identified by the prefix “FG” followed by their assigned number (FG1–FG4). These identifiers were used consistently when reporting verbatim quotations, allowing the source of each quotation to be distinguished while preserving participant anonymity.

ATLAS.ti^®^ (version 25.0.1; Scientific Software Development GmbH, Germany), including its AI-assisted conversational functionality, was used to support data management and analysis in both Phase 1 and Phase 2. Only anonymised transcripts were imported into ATLAS.ti^®^ or processed using its AI-assisted functionality. The AI-assisted tool generated preliminary suggestions for descriptive codes and their organisation into analytical categories; it did not independently determine the final codes, categories, themes, or interpretations. Because the generation of preliminary codes and categories may itself influence the interpretative process, these automatically generated outputs were treated as provisional analytical suggestions requiring researcher review rather than as established analytical findings. Each suggestion was individually reviewed against the corresponding transcript data by the research team. During this researcher-led review, suggested codes and categories could be retained, modified, merged, renamed, or removed when considered redundant, overly broad, insufficiently grounded in participants’ accounts, or interpretatively inappropriate. Researchers also created additional codes manually when relevant meanings in the transcripts were not adequately represented by the AI-generated suggestions. The final coding frameworks, themes, and interpretations were therefore determined by the research team through interpretative engagement with the original data rather than by the AI-assisted tool.

#### 2.5.1. Phase 1. Individual Interviews

The interviews were transcribed using TurboScribe^®^ (web-based transcription service; TurboScribe, Inc., Bellevue, Washington, USA; https://turboscribe.ai/ accessed November 2025) and manually reviewed, corrected, and verified for accuracy. An inductive thematic analysis was then conducted, allowing codes, categories, and themes to be developed from participants’ accounts rather than from a predetermined analytical framework.

The researcher-led analysis was undertaken by one researcher (H.G.-d.l.T.) and subsequently reviewed by a second researcher (C.-A.R.-S.), who examined the consistency of the coding process and the appropriateness of the allocation of codes to categories. Any discrepancies were resolved through discussion until consensus was reached.

Informational sufficiency was assessed during the analysis and considered separately from the a priori sampling target. The research team examined the recurrence of meanings and codes across participants’ accounts, the emergence of new codes and categories as the analysis progressed, the diversity of perspectives represented, and whether the data provided sufficient experiential material to address the research objectives. On this basis, the individual interview dataset was considered informationally sufficient to support the interpretative analysis and address the study objectives.

A descriptive co-occurrence matrix was generated from the coded data to explore patterns of co-occurrence among the subthemes identified during the inductive analysis. Co-occurrences were calculated at the code level, with a co-occurrence defined as the presence of codes belonging to different subthemes within the same meaning unit (MU). Code-level co-occurrence frequencies were subsequently aggregated according to the subthemes to which the respective codes belonged to construct the descriptive matrix. Co-occurrence frequencies were used solely as a complementary descriptive mapping tool to identify patterns warranting closer interpretative examination. No predefined thresholds or ordinal categories were applied to the co-occurrence frequencies, which were interpreted descriptively as patterns within the coded material rather than as measures of the strength or statistical significance of relationships between subthemes. Consistent with the hermeneutic phenomenological orientation of the study, interpretation remained grounded in participants’ accounts, the contextual meaning of the co-occurring coded segments, and the researchers’ interpretative engagement with the data. The thematic findings and descriptive co-occurrence patterns were considered together in the interpretative synthesis of Phase 1, which identified the principal dimensions of students’ experiences and informed the initial analytical framework for Phase 2.

The Phase 1 interpretative findings also informed the focus group guide, which was designed to further explore, refine, and expand the identified themes and subthemes and to facilitate discussion of potential improvements to the clinical placement programme. Five preliminary improvement proposals derived from the Phase 1 findings were included as prompts to stimulate discussion rather than as predetermined analytical categories.

#### 2.5.2. Phase 2. Focus Group

Phase 2 adopted a directed deductive thematic analysis, using the themes and subthemes identified in Phase 1 as the initial analytical framework. After familiarisation with the transcript, the discourse was segmented into MUs and coded against that framework. MUs that were not adequately represented by the initial framework were retained and examined to determine whether they reflected additional meanings, elaborations, or divergent perspectives rather than being forced into existing categories. For each subtheme, the research team compared the focus group discourse with the Phase 1 findings and examined whether participants’ accounts converged with previously identified meanings, elaborated or nuanced those meanings, or expressed divergent or contradictory perspectives.

Convergent accounts were used to identify meanings shared across the two phases, whereas elaborations and nuances were incorporated into the interpretation and operational definitions where appropriate. Divergent or contradictory accounts were retained as analytically meaningful findings and used to represent variation in participants’ experiences rather than being resolved or excluded. Relationships between subthemes were examined to construct interpretative axes, improvement proposals discussed and elaborated during the focus group were identified and organised thematically, and representative verbatim quotations were selected to ensure traceability between the data and the interpretation.

The purpose of this second analytical phase was to understand how the group discourse converged with, elaborated, nuanced, or challenged the findings from the individual interviews, rather than to validate or confirm them. The integration of the inductive findings from Phase 1 with the deductively analysed focus group data in Phase 2 provided a more comprehensive interpretation of the phenomenon while allowing convergent, elaborative, and divergent perspectives to remain visible. The Phase 2 analysis was conducted jointly by three members of the research team (Z.S.-C., H.G.-d.l.T., and C.-A.R.-S.).

All 14 participants were invited to review their own transcripts for accuracy, and all completed this review. No transcription errors were identified, and no corrections or changes were requested. Participants did not review or provide feedback on the final themes or interpretations.

### 2.6. Trustworthiness

The four criteria of Lincoln and Guba’s trustworthiness framework [40] were considered throughout the study.

Dependability was addressed by retaining records of the study procedures and key methodological and analytical decisions, including the interview and focus group guides, verbatim transcripts, field notes from the focus group, the final coding framework, and the organisation of codes into subthemes and themes. The same interview guide was used for all individual interviews to maintain procedural consistency, whereas the focus group followed a separate guide developed from the Phase 1 findings and was complemented by field notes documenting group dynamics. Analytical coherence was addressed by maintaining traceability between participants’ accounts, codes, subthemes, themes, and the resulting interpretations. However, no formal reflexive journal was maintained, and sufficiently detailed records of the initial AI-generated outputs and subsequent decisions at the level of individual codes were not retained, as acknowledged in the Research Team and Reflexivity and Limitations sections.

Credibility was addressed through the sequential use of individual interviews and a focus group, which enabled the Phase 1 findings to be further explored through collective discussion, as well as through verbatim transcription of all recordings, the inclusion of representative quotations illustrating the thematic findings, and participants’ review of their own transcripts for accuracy. All 14 participants completed this transcript review, and no transcription errors were identified, and no corrections were requested. During data collection, participants were encouraged to describe their experiences freely, with the interviewer using active listening and avoiding unnecessary interruption. Traceability was maintained by preserving a clear link between participants’ original accounts and the interpretations presented.

Confirmability was addressed through researcher review of the analytical process and explicit consideration of convergent, elaborative, divergent, and contradictory perspectives. During data collection, the interviewer sought to avoid leading participants’ accounts, while the potential influence of the researchers on data generation and interpretation was explicitly recognised and considered through reflexive consideration and discussion within the research team.

Finally, transferability was addressed by providing a detailed description of the study setting, participants’ characteristics, and the structure of clinical placements within the Nursing Degree curriculum. This contextual information was provided to enable readers to assess the potential relevance of the findings to other educational settings with similar characteristics.

### 2.7. Ethical Considerations

The study was conducted in accordance with the Declaration of Helsinki and was approved by the Research Ethics Committee (CEI/CEIm) of Las Palmas (Code: 2025-389-1; Date: 19 August 2025). All participants received information about the purpose and procedures of the study and provided written informed consent before participation. Participation was entirely voluntary, and participants were informed that they could decline to participate or withdraw at any time without academic or personal consequences.

Confidentiality was maintained throughout data collection, analysis, and reporting. Before being imported into ATLAS.ti or processed using its AI-assisted functionality, the transcripts were anonymised by removing participants’ names and replacing them with coded identifiers. Audio recordings were uploaded directly to TurboScribe^®^ for transcription, after which the transcripts were stored on the ULPGC server with access restricted to the principal investigator. Following transcription, the audio files were deleted from TurboScribe^®^ and from the devices used for recording. A copy of the original audio recordings is retained on a device under the custody of the principal investigator for two years, in accordance with the approved data-management procedures, after which it will be permanently deleted. Access to the remaining research data was restricted to the research team, and the findings were reported without personally identifying information. 

The use of TurboScribe^®^ for transcription and ATLAS.ti^®^, including its AI-assisted functionality, was explicitly specified in the research protocol submitted for ethical review and formed part of the approved methodological and data-management procedures.

## 3. Results

### 3.1. Phase 1: Results of Individual Interviews

#### 3.1.1. Sociodemographic Characteristics of the Participants

The sociodemographic and academic characteristics of the participants are presented in Table 1. The Phase 1 sample comprised 10 nursing undergraduates, including four third-year and six fourth-year students, aged 20–25 years. Most participants were female, and none were employed at the time of data collection. Regarding previous educational background, two participants had completed prior studies in the health sciences, whereas the remaining participants had either completed studies in unrelated fields or had entered the degree programme directly after upper secondary education.

#### 3.1.2. Development of the Thematic Framework

The inductive analysis of the interview transcripts generated 15 subthemes, which were organised into five overarching themes. Table 2 presents the thematic framework, including the themes, subthemes, associated codes, and their operational definitions. This framework constituted the analytical basis for the deductive analysis undertaken in Phase 2.

Although presented separately for analytical clarity, the themes were closely interconnected and collectively described students’ experiences of clinical placements from relational, educational, organisational, and assessment perspectives.

#### 3.1.3. Thematic Findings

- General Experience

Participants generally described their clinical placements as a predominantly positive educational experience, although these evaluations were frequently accompanied by reservations related to specific placements or organisational aspects. Their accounts associated satisfaction with the learning opportunities available, the clinical environment, and the extent to which placements corresponded with their expectations.

*“Well, I think they have been quite good… although I had a bad experience.”*

(P1)

Negative experiences were mainly associated with placements perceived as offering limited learning opportunities, and participants described resulting feelings of insecurity and insufficient professional preparedness.

*“I think it has quite a few shortcomings… I am going to finish the degree, and I feel that I know nothing, and that makes you feel very insecure.”*

(P4)

Students also reflected on the discrepancy between their expectations before entering clinical practice and the realities they encountered. For some, initial uncertainty gradually changed as they experienced different clinical settings.

*“…At the beginning I was like, oh, maybe if this is going to be like this, then I do not like it.”*

(P3)

- Relationship with Staff

Relationships with nursing staff were consistently prominent in participants’ accounts. Participants associated feeling welcomed, supported, and recognised within the team with greater confidence and opportunities to participate in patient care.

*“Normally the treatment was very positive… they were very patient with me…”*

(P2)

Conversely, some participants described experiences of exclusion, disrespect, or discrimination that they associated with poorer emotional well-being and a reduced sense of belonging.

*“…when I was transitioning… they said, you are trans, right?* *…* *comments that were completely out of place.”*

(P9)

- Autonomy and Learning

Students generally described greater autonomy as they progressed through the degree programme. Participants associated increasing responsibility with greater confidence and opportunities for learning.

*“…now in fourth year, I think that the autonomy we have is much greater…”*

(P1)

Learning opportunities were particularly valued when students were able to participate actively in clinical care rather than remain passive observers. Views regarding competency assessment were more heterogeneous, with several participants questioning the objectivity of assessment procedures.

*“…it depends a bit on how you write it… whether you do it well or not…”*

(P5)

Some students also suggested that participation in longer shifts and on-call duties could provide additional opportunities to consolidate clinical competence.

*“To be able to do a morning and an afternoon shift… I do not see the point that Erasmus students… are allowed to do on-call duties and nights.”*

(P6)

- Structure and Organisation

Participants expressed differing views regarding rotation length. Some considered short rotations insufficient for adaptation and learning, particularly in highly specialised settings.

*“Sometimes the amount of time we spend in a service is too short…”;*

(P8)

*“…I would extend the duration in the ICU or emergency department…”*

(P2)

Conversely, others considered that prolonged rotations restricted exposure to different clinical settings and limited the breadth of their educational experience.

*“…rotations should be shortened, … in the end that is what makes the student learn.”*

(P9)

Participants also described limited exposure to specialised clinical settings and suggested greater flexibility in placement allocation and broader exposure to clinical areas they considered important for professional preparation.

*“…there are many specialised services about which we know absolutely nothing.”*

(P7)

*“I think what I would mainly change is to make things easier…”;*

(P8)

*“…every fourth-year student should go through ICU and emergency department.”*

(P4)

- Assessment of Clinical Placements

Participants questioned whether written assessment documents adequately reflected their clinical performance and expressed concerns about the subjectivity of these methods.

*“I think those things [assessment documents]… do not really show the student’s work.”*

(P8)

Reflective assignments were frequently described as time-consuming and of limited educational value.

*“…I would remove them, yes, because they take time away from us…”*

(P10)

Several participants also described inconsistencies in marking and perceived that assessment could vary according to the assessor.

*“…one gives me a 6 and the other a 10. I think it is very subjective.”*

(P7)

Several participants expressed a preference for greater direct observation of clinical performance and feedback from clinical tutors rather than predominant reliance on written documentation.

*“I cannot have a tutor who does not see my face throughout the whole service and then gives me a grade out of nowhere.”*

(P4)

#### 3.1.4. Descriptive Co-Occurrence Patterns Between Subthemes

The descriptive co-occurrence analysis provided a complementary overview of patterns identified within the coded material (Table 3). Co-occurrences represented the frequency with which codes belonging to different subthemes occurred within the same MU. The most recurrent code-level co-occurrence patterns involved satisfaction with clinical placements and codes corresponding to treatment by nursing staff, inclusion in the team, and learning opportunities. Recurrent patterns were also observed among codes corresponding to autonomy, learning, and assessment-related subthemes.

These frequencies were interpreted descriptively and used solely to identify co-occurrences warranting closer interpretative examination, rather than as measures of the strength or statistical significance of relationships between subthemes. Their meaning was interpreted in conjunction with the thematic findings and the contextual examination of participants’ accounts.

#### 3.1.5. Interpretative Synthesis of Phase 1 Findings

Considering the thematic findings alongside the descriptive co-occurrence patterns as a complementary mapping tool, the interpretative synthesis suggested that students’ experiences of clinical placements were organised around three interrelated dimensions: the relational environment, opportunities for learning and autonomy, and the organisation and assessment of clinical placements. Participants’ accounts linked the relational environment with their experiences of participation and learning, while progressive autonomy was described in relation to the supervisory context. Organisational and assessment-related aspects were also related to participants’ perceptions of the educational value of their placements.

Overall, these three dimensions provided an integrated representation of the meanings identified across the individual interviews and formed the initial analytical framework and discussion guide used in Phase 2.

### 3.2. Phase 2: Results of Focus Group

#### 3.2.1. Sociodemographic Characteristics of the Focus Group Participants

The focus group comprised four fourth-year nursing students, all female and aged 21 years. None of the participants were employed during the study period, and all had entered the Nursing Degree after completing upper secondary education, resulting in a relatively homogeneous educational background. Their characteristics are presented in Table 4.

#### 3.2.2. Comparison and Refinement of the Phase 1 Subthemes in the Focus Group

Building on the Phase 1 interpretative synthesis, the focus group analysis examined whether participants’ accounts converged with previously identified meanings, elaborated or nuanced them, or expressed divergent or contradictory perspectives. Overall, participants’ accounts largely converged with the Phase 1 thematic structure while also providing further elaboration and qualification of several subthemes. Divergent or contradictory perspectives were retained in the analysis where present. No entirely new overarching themes were identified in the focus group.

Table 5 summarises the comparison between the two phases and the resulting integrated definitions of the subthemes.

#### 3.2.3. Integrated Findings Across the Two Phases

Across the two phases, participants’ accounts encompassed three interrelated dimensions: the relational environment, opportunities for learning and autonomy, and the organisation and assessment of clinical placements. The focus group provided additional collective perspectives on these dimensions without substantially altering the thematic structure developed from the individual interviews. No entirely new overarching themes were identified, although several Phase 1 subthemes were further elaborated or nuanced, while divergent or qualifying perspectives were retained where present, reflecting variation in participants’ experiences.

#### 3.2.4. Improvement Proposals Discussed in the Focus Group

The focus group also elaborated contextually grounded suggestions for potential improvements to the clinical placement programme. These proposals are summarised by theme in Table 6 and should be understood as perspectives developed through collective discussion rather than as recommendations for implementation. 

#### 3.2.5. Representative Verbatim Quotations

Table 7 presents representative verbatim quotations illustrating the Phase 2 findings. Quotations were selected to maintain traceability between participants’ accounts and the analysis and, where present in the data, to represent divergent, less frequent, or qualifying perspectives alongside more recurrent accounts.

## 4. Discussion

The findings of this study provide an integrated understanding of how nursing students experience clinical placements, suggesting that learning and satisfaction are related to the interaction of relational, organisational, educational, and assessment-related factors rather than to the formal structure of the curriculum alone. This interpretation is consistent with the literature, which describes the clinical experience as being shaped by the learning environment, relationships with staff, the degree of autonomy granted, and the assessment systems used [1,9,10].

In addition, the coexistence of satisfaction and emotional distress described by the participants, as well as the progressive adjustment of their initial expectations, is consistent with studies linking the quality of the clinical experience to students’ future career decisions and suggesting that unfavourable experiences may contribute to students reconsidering their preferred specialty or even their continuation in the profession [5,6,7,8,41].

This pattern also connects with the literature on the so-called “reality shock” experienced by nursing students when facing a clinical environment that does not match what they had anticipated. This phenomenon has been associated with anxiety, loss of confidence, and, in more pronounced cases, withdrawal from the programme [41,42]. In this regard, the accumulated fatigue and feelings of insufficiency described by some participants are consistent with recent evidence on the high prevalence of academic burnout among nursing students and its association with conditions within the clinical learning environment rather than with purely academic workload [43,44,45]. Taken together, these findings and the existing literature suggest that emotional distress during clinical placements warrants attention when considering strategies for early identification and support during the Practicum modules. The coexistence of supportive and unsupportive experiences may also contribute to the way students progressively construct and reconsider their emerging professional identity throughout the programme.

A prominent theme in participants’ accounts concerned their relationships with staff in the clinical units and services. When interpreted alongside previous research, these findings support the relevance of the relational climate to students’ clinical experiences and are consistent with belongingness as an important component of participation and learning in clinical settings [3,4,14,46]. However, the present findings provide an important nuance: inclusion and rejection were not experienced as opposite and stable categories. Rather, participants’ accounts suggest a continuum through which the same student may move depending on the specific clinical setting and supervisory relationships encountered during each rotation. This interpretation is consistent with a dynamic understanding of belongingness as a relational process that may vary across placements rather than as a stable characteristic of a particular clinical environment. From an educational perspective, this may support consideration of the preparation and suitability of staff involved in student supervision when reviewing clinical placement arrangements.

Several studies indicate that incivility and rejection towards nursing students are not exceptional phenomena in clinical placement settings but are relatively frequent and are associated with stress, poorer mental health, and intention to leave the profession [23,24,25,47]. Although such experiences were not widely reported in our study, one participant specifically described discrimination related to gender identity. Although the literature describes how gender stereotypes traditionally associated with the nursing profession may shape the experiences of those who do not fit within them [48,49], there are still few studies documenting stigma and discrimination towards LGBTQI+ nursing students [50]. Considered alongside this literature, the experience identified in the present study suggests that institutional approaches to welcoming and supporting students in clinical placements could benefit from explicitly considering sexual and gender diversity. However, this represents a broader implication derived from the interpretation of the findings and existing evidence rather than a specific recommendation directly expressed by participants.

Another prominent theme concerns students’ autonomy and the role of tutors. The progressive assumption of responsibility can be interpreted in relation to models of experiential learning in which experience, reflection, and conceptualisation require appropriate support to facilitate learning [51,52]. Participants’ accounts indicated that autonomy was not always experienced as a gradual process linked to competence, but rather varied considerably according to the clinical context, the professionals supervising them, and the opportunities available for participation. When considered alongside the literature, this variability raises the possibility that students may experience either insufficient opportunities for autonomous practice or levels of responsibility that they perceive as exceeding the supervision available to them. These findings suggest that more structured approaches to progressive supervision may warrant consideration, while the specific form that such approaches should take requires evaluation within each educational and clinical context. Participants’ concerns regarding the limited presence or continuity of some tutors further highlight supervision as an area warranting attention in future reviews of the clinical placement programme.

Participants’ accounts regarding the structure and organisation of clinical placements offer opportunities for comparison with the literature. Their differing perspectives on rotation duration are consistent with previous research showing that the educational implications of placement length may vary according to the characteristics of the clinical setting. Although longer rotations have been associated with better relationships with tutors and a more favourable perception of the environment, excessive duration may generate monotony and perceived stagnation in learning [30,31,32]. Participants specifically suggested adapting rotation length to the characteristics and complexity of clinical settings. Considered alongside previous evidence, this suggests that greater flexibility in rotation duration may warrant exploration rather than the application of a uniform criterion across all placements [33,34]. However, the present findings do not establish an optimal duration or support a specific rotation model. Participants also proposed linking clinical placements to specific subjects and ensuring rotation through key units such as intensive care or emergency departments, or through central areas such as paediatrics. Likewise, participants’ preference for greater flexibility in choosing placement centres to reduce travel costs and time is consistent with studies that identify preference for placement location as one of the organisational factors influencing student satisfaction and even future professional perspectives [5].

Finally, in the assessment domain, participants questioned the perceived subjectivity of reflective documents and their correspondence with actual clinical performance. These concerns can be interpreted in light of the broader literature on clinical competency assessment. Written reflection has been reported to support critical thinking and the tutor–student relationship, although important limitations have also been identified, including workload and inter-rater variability when grading depends more on the narrative quality of the document than on direct observation of clinical performance [20,26,27,28,29]. Although concerns about inter-rater reliability are not exclusive to reflective documents and have also been reported for more structured assessment methods, such as the Objective Structured Clinical Examination (OSCE) [53,54], participants themselves primarily advocated greater direct observation of clinical performance and more continuous feedback rather than any specific alternative assessment instrument. Considered together with the literature, their concerns suggest that more programmatic and balanced approaches combining several sources of evidence—such as direct observation, portfolios, and structured feedback—may merit consideration [55]. OSCE-type assessment may represent an additional option for selected competencies, but this is an author-derived educational implication informed by the literature rather than a proposal arising directly from participants’ accounts.

An important contribution of this study is the integrated interpretation generated across the two phases. The findings suggest that the relational environment, opportunities for learning and autonomy, and the organisation and assessment of clinical placements are interconnected aspects of students’ experiences rather than isolated components. Considered in relation to previous research, which has frequently examined these aspects separately, the present study provides a framework for understanding how they may interact within students’ lived experiences of clinical placements. This framework should be regarded as an interpretative account grounded in this specific sample and context rather than as evidence of causal relationships between these dimensions.

The practical implications of these findings should be interpreted cautiously given the qualitative design, the small self-selected sample, and the single-campus setting. Several potential improvements were discussed and elaborated during the focus group, including greater flexibility in placement organisation, rotation lengths adapted to the characteristics and complexity of clinical settings, broader exposure to key clinical areas, greater continuity and support in clinical supervision, and greater emphasis on direct observation and feedback in assessment. These suggestions reflect participants’ perspectives developed through collective discussion and identify areas that may warrant consideration in future curricular reviews rather than prescriptive recommendations for implementation.

Other potential strategies represent broader educational implications developed by the authors through interpretation of the findings in conjunction with the existing literature. These include more balanced competency-assessment models incorporating different sources of evidence and, where appropriate, OSCE-type assessment for selected competencies; more structured institutional approaches to tutor preparation and student supervision; explicit institutional measures addressing incivility and discrimination; and psychological or peer-support mechanisms during clinical placement periods. These strategies should be regarded as options for further consideration and evaluation rather than as recommendations directly derived from participants’ accounts. Their feasibility, acceptability, and effectiveness would need to be assessed within the institutional context and in broader and more diverse student populations.

One of the strengths of this study is its sequential design, which provided both individual and collective perspectives and enabled potential improvements to clinical placements to be discussed and further elaborated through collective reflection, in line with current recommendations to involve students as active agents in curricular improvement rather than merely as informants [56]. These proposals were organised according to the previously established themes and subthemes, maintaining analytical coherence between the issues identified and the areas discussed for improvement. Furthermore, the combination of inductive and directed deductive thematic analyses enabled the framework developed during the interview phase to be revisited and further elaborated through collective discussion in the focus group, while retaining convergent, nuanced, and divergent perspectives within the integrated analysis.

Nevertheless, several limitations should be acknowledged. The focus group was small (*n* = 4) and consisted exclusively of fourth-year female students, although its size was consistent with the lifeworld methodological approach adopted [36]. Although 15 additional students expressed interest, they were unable to participate because of scheduling constraints. This limits the transferability of this second phase to other student profiles, especially third-year students or male students. The relatively short duration of the individual interviews (mean approximately 14 min; range: 9–23 min) may also have limited the depth with which some experiences and meanings were explored, despite the use of probing questions and participants’ direct experience of the phenomenon under study. In addition, individual interviews were conducted using two different modes (five face-to-face and five online via Microsoft Teams). Although the same semi-structured interview guide was used in both modes, differences in the interactional context may have influenced the nature or depth of participants’ accounts.

Because both the focus group guide and the directed deductive analysis were structured around the themes and subthemes identified in Phase 1, the questions and subsequent analytical framework may have oriented the collective discussion towards previously identified meanings and limited the emergence of alternative interpretations or entirely new categories. Although the guide was used flexibly, participants were encouraged to introduce alternative perspectives or issues not represented in the initial framework, and MUs not adequately represented by that framework were retained for further examination. Nevertheless, the possibility of framework-related constraints cannot be excluded.

The use of AI-assisted functionality during the qualitative analysis represents an additional methodological consideration. In both phases, AI-assisted output was used as an analytical aid, and all AI-generated suggestions were reviewed against the original anonymised data by the research team. Nevertheless, this analytical support may have influenced aspects of the coding and analytical process. In addition, sufficiently detailed records of the initial AI-generated outputs and subsequent decisions made at the level of individual codes were not retained, limiting the verifiability and reproducibility of this part of the analytical process. The final codes, categories, themes, and interpretations were determined by the research team through researcher-led engagement with the original data. Furthermore, participants reviewed their own transcripts for accuracy but did not review or provide feedback on the final themes or interpretations. Consequently, transcript checking should not be interpreted as interpretative member checking, and the final analytical framework reflects the researchers’ interpretation of participants’ accounts.

The recruitment procedure may also have introduced self-selection bias, as participation depended on students responding voluntarily to either face-to-face or institutional email invitations from the principal researcher. Students who were particularly motivated or who held more firmly established views about clinical placements may therefore have been more likely to participate, potentially leaving the perspectives of less engaged students underrepresented. In addition, the principal researcher had a pre-existing peer relationship with some fourth-year participants. Although she had no teaching, supervisory, assessment, or other hierarchical role in relation to them, and procedural and reflexive strategies were used to address potential researcher influence, this prior relationship may have affected participants’ willingness to disclose critical or negative experiences or contributed to socially desirable responses. This potential influence cannot be completely excluded and should therefore be considered when interpreting the findings. Finally, as this was a single-centre study conducted at one campus of the ULPGC, caution is required when transferring these findings to other nursing programmes with different organisational or curricular characteristics.

These findings also open several avenues for future research. Quantitative or mixed-methods studies using validated clinical learning environment instruments in representative samples from all ULPGC sites and academic years could examine the phenomena identified here in a broader population. Longitudinal studies following the same cohort throughout the five Practicum modules could explore how autonomy, satisfaction, and relationships with staff evolve as students progress through the degree. Further research could also compare different approaches to clinical competency assessment—including reflective documents, structured observation, and OSCEs—in terms of inter-rater reliability and perceived fairness among students. Given increasing pressure on the availability of certain clinical placements, the potential role of high-fidelity simulation as a complement to rotations in particularly high-demand units and services also warrants investigation. Finally, future studies should evaluate the feasibility, acceptability, and potential effects of selected improvement proposals discussed during the focus group and broader educational strategies arising from these findings on students’ learning experiences, satisfaction, and perceived quality of clinical placements.

## 5. Conclusions

In this qualitative, single-site study, nursing students’ accounts suggested that their clinical placement experiences were shaped by the interaction of relational, organisational, educational, and assessment-related dimensions. Relationships with clinical staff, inclusion within the clinical team, opportunities for learning and progressive autonomy, and the availability of supervision emerged as particularly relevant aspects of these experiences. Participants also identified challenges related to placement organisation and assessment, including rotation characteristics and the perceived correspondence between written assignments and clinical performance. 

These context-specific findings may inform future reviews of the clinical placement programme by incorporating students’ perspectives into curricular reflection. The focus group enabled potential improvements concerning supervision, placement organisation, and assessment to be discussed and elaborated through collective reflection. These proposals should be regarded as areas for further consideration rather than as recommendations for implementation, and their feasibility, acceptability, and effectiveness require further evaluation. Given the qualitative design, self-selected sample, and single-campus setting, the transferability of the findings to other nursing programmes should be considered with caution and explored in larger and more diverse samples.

## Figures and Tables

**Table 1:** Characteristics of participants in Phase 1.

Participant	Gender	Age (Years)	Academic Year	Employment Status	Previous Educational Background
P1	Female	23	Fourth	Not employed	Higher Vocational Diploma in Clinical Laboratory Science
P2	Female	21	Fourth	Not employed	Upper secondary education
P3	Male	23	Fourth	Not employed	Upper secondary education
P4	Female	25	Third	Not employed	Bachelor’s degree in Criminology
P5	Female	21	Fourth	Not employed	Upper secondary education
P6	Female	21	Third	Not employed	One year of Physiotherapy studies
P7	Male	20	Third	Not employed	Upper secondary education
P8	Female	21	Fourth	Not employed	Upper secondary education
P9	Female	22	Third	Not employed	Higher Vocational Diploma
P10	Female	23	Fourth	Not employed	Higher Vocational Diploma in Medical Imaging

P: Participant.

**Table 2:** Thematic framework derived from the inductive analysis.

Theme	Subthemes	Associated Codes	Operational Definition
General Experience	Satisfaction with clinical placements	Satisfaction; Gratitude; Acceptance; Security; Overcoming	Overall positive evaluations of clinical placements, characterised by feelings of well-being, confidence, acceptance, and personal growth arising from the educational experience.
	Negative experiences	Abuse of power; Ambiguity; Silence; Social pressure; Fatigue; Burnout; Feelings of insufficiency; Discontent	Adverse experiences during clinical placements associated with emotional distress, psychological exhaustion, or negative perceptions of the educational environment.
	Comparison with expectations	Initial expectations; Theory–practice comparison; Professional reality; Frustration; Self-criticism	Discrepancy between students’ prior expectations and the reality experienced in the clinical practice context.
Relationship with Staff	Treatment by nursing staff	Respect; Responsibility; Patient care; Quality of care; Professional support	Perceptions of the quality of interactions with nursing staff, including relational, ethical, and professional aspects.
	Inclusion in the team	Inclusion; Integration into the team; Interpersonal relationships; Social support; Emotional support	Experiences related to students’ sense of belonging to the clinical team and the support received, as perceived in relation to learning and active participation.
	Discrimination or rejection	Discrimination; Rejection; Lack of respect; Abuse of power; Silence	Experiences of exclusion, unequal treatment, or negative attitudes perceived during clinical placements.
Autonomy and Learning	Level of autonomy	Autonomy; Self-efficacy; Decision-making; Responsibility; Adaptation	Degree of students’ progressive independence in performing clinical tasks and assuming professional responsibilities.
	Learning opportunities	Learning; Practical learning; Experiential learning; Skills development	Opportunities for acquiring practical and clinical skills during clinical placements.
	Assessment of competencies	Assessment of competencies; Subjectivity; Self-assessment; Self-reflection; Feelings of insufficiency	Perceptions of the assessment of clinical competencies and the emotions and reflections associated with this process.
Structure and Organisation	Duration of rotations	Insufficient duration; Time burden; Adaptation to the environment; Routine	Perceived adequacy of the duration of clinical rotations and its relationship with students’ adaptation and learning experiences.
	Variety of clinical settings	Variety of services; Monotony; Limited experiences; Variability in workload	Perceptions of the diversity—or lack thereof—of the clinical settings in which placements were undertaken.
	Proposals for improving planning	Proposals for change; Suggestions; Data collection; Personal priorities	Students’ suggestions aimed at improving the planning, organisation, and distribution of clinical placements.
Assessment of Clinical Placements	Assessment methods	Assessment methods; Institutional assessment; Subjectivity	Perceptions of the systems and procedures used to assess clinical placements and their perceived effectiveness.
	Reflective assignments	Reflective documents; Reflection; Reflection on practice; Self-reflection	Perceptions of the usefulness of reflective assignments as tools for learning and assessment.
	Perceived fairness in assessment	Fairness/unfairness; Comparison between students; Subjectivity; Abuse of power	Perceptions of fairness or arbitrariness in the assessment process, including comparisons between students and experiences perceived as inequitable.

**Table 3:** Descriptive co-occurrence matrix of the subthemes identified in Phase 1.

Subtheme	A	B	C	D	E	F	G	H	I	J	K	L	M	N	O
A. Satisfaction with clinical placements	—	4	6	12	10	2	9	11	5	3	4	2	5	2	2
B. Negative experiences		—	9	3	5	8	4	3	8	7	6	9	4	2	10
C. Comparison with expectations			—	4	3	3	6	5	7	4	3	5	2	1	6
D. Treatment by nursing staff				—	11	7	10	9	4	2	3	2	3	1	2
E. Inclusion in the team					—	6	7	6	5	2	3	3	3	1	4
F. Discrimination or rejection						—	2	2	2	1	2	4	1	1	3
G. Level of autonomy							—	11	8	6	5	4	4	2	5
H. Learning opportunities								—	6	4	7	3	5	2	3
I. Assessment of competencies									—	5	4	9	4	5	8
J. Duration of rotations										—	6	3	7	2	3
K. Variety of clinical settings											—	2	8	1	2
L. Proposals for improving planning												—	4	8	11
M. Assessment methods													—	3	3
N. Reflective assignments														—	6
O. Perceived fairness in assessment															—

Note: Letters A–O correspond to the subthemes listed in the first column.

**Table 4:** Characteristics of the focus group participants.

FG Participant	Gender	Age (Years)	Academic Year	Employment Status	Previous Educational Background
FG1	Female	21	Fourth	Not employed	Upper secondary education
FG2	Female	21	Fourth	Not employed	Upper secondary education
FG3	Female	21	Fourth	Not employed	Upper secondary education
FG4	Female	21	Fourth	Not employed	Upper secondary education

FG: focus group.

**Table 5:** Comparison and refinement of the Phase 1 subthemes through the focus group.

Theme	Subtheme	Evidence from the Focus Group	Relationship to Phase 1 Findings	Integrated Interpretative Definition
General Experience	Satisfaction with clinical placements	Positive experiences were mainly associated with autonomy, inclusion, and supportive relationships with staff.	Convergent	Participants associated overall satisfaction with integration within the clinical team and active participation in patient care.
	Negative experiences	Accounts emphasised mistreatment, anxiety, emotional distress, and demotivation.	Convergent with further elaboration	Includes emotional distress described in relation to negative interpersonal experiences and organisational limitations during clinical placements.
	Comparison with expectations	Participants described a progressive mismatch between initial expectations and clinical reality.	Convergent with further elaboration	Expectations evolved throughout the programme as students contrasted their initial assumptions with their clinical experiences.
Relationship with Staff	Treatment by nursing staff	Respectful interactions promoted learning, whereas disrespectful attitudes limited participation.	Convergent	Participants’ accounts associated the quality of interactions with nursing staff with their overall clinical placement experiences.
	Inclusion in the team	Feelings of inclusion varied according to individual professionals and clinical settings.	Convergent	Participants perceived their inclusion within the clinical team as varying according to the attitudes and behaviours of individual professionals as well as the organisational context.
	Discrimination or rejection	Participants described experiences of exclusion, invalidation, and discriminatory attitudes.	Convergent with further elaboration	Includes explicit and implicit forms of exclusion that negatively affected students’ sense of belonging.
Autonomy and Learning	Level of autonomy	Experiences ranged from excessive responsibility to insufficient autonomy depending on the placement context.	Convergent with further elaboration	Participants perceived autonomy as inconsistent across placements and associated these differences particularly with supervisors’ attitudes and the clinical context.
	Learning opportunities	Participants associated opportunities for learning with supervision, trust, and active participation in care.	Convergent	Participants associated learning opportunities with the relational and supervisory context of each placement.
	Assessment of competencies	Participants questioned whether assessment reflected actual clinical competence.	Convergent with further elaboration	Participants perceived a mismatch between formal assessment procedures and observed clinical performance.
Structure and Organisation	Duration of rotations	Participants argued that rotation length should reflect the complexity of the clinical setting.	Convergent with further elaboration	Participants perceived the educational value of different rotation lengths as varying according to the characteristics and complexity of the clinical setting.
	Variety of clinical settings	Participants highlighted limited exposure to several specialised clinical areas.	Convergent	Greater diversity of clinical settings was perceived as important for comprehensive professional preparation.
	Proposals for improving planning	Numerous suggestions focused on placement allocation, flexibility, and compulsory exposure to key services.	Convergent with further elaboration	Collective discussion further elaborated proposals concerning the planning and organisation of clinical placements.
Assessment of Clinical Placements	Assessment methods	Participants considered that written assessments did not adequately reflect clinical performance.	Convergent	Assessment methods were perceived as relying excessively on written documentation rather than direct observation of practice.
	Reflective assignments	Reflective assignments were frequently viewed as repetitive and disconnected from clinical learning.	Convergent with further elaboration	Reflective assignments were perceived as having limited educational value when not linked to observed clinical performance.
	Perceived fairness in assessment	Participants questioned the consistency and fairness of assessment decisions.	Convergent	Participants associated perceived fairness in assessment with direct supervision, transparency of assessment criteria, and consistency between assessors.

**Table 6:** Improvement proposals discussed and elaborated through the focus group.

Theme	Improvement Proposals Discussed in the Focus Group
General Experience	Greater integration between theoretical teaching and clinical placements; earlier clinical exposure; and opportunities for all students to rotate through key clinical settings, including intensive care and emergency departments.
Relationship with Staff	Enhanced training of clinical tutors in mentoring, communication, and supportive supervision; and greater recognition of the educational role of clinical staff involved in student supervision.
Autonomy and Learning	More structured supervision to support progressive autonomy; greater continuity through assignment to the same clinical tutor where possible; and increased opportunities for supervised participation in clinical procedures.
Structure and Organisation	Rotation lengths adapted to the characteristics and complexity of clinical settings; greater exposure to specialised clinical areas; and increased flexibility and transparency in placement allocation.
Assessment of Clinical Placements	Greater use of direct observation of clinical performance and structured feedback; and revision of reflective assignments, including the Standardised Care Plan, to better align them with clinical practice and competence assessment.

**Table 7:** Representative verbatim quotations illustrating the themes and subthemes identified in the focus group.

Theme	Subtheme	Representative Verbatim Quotations
General Experience	Satisfaction with clinical placements	“In the placements I have had, I have felt very integrated by all the staff and satisfied.” (FG3)
	Negative experiences	“These were very stressful situations that increased my anxiety. For the first time, I did not feel like going to clinical placements.” (FG2)
	Comparison with expectations	“I started with lower expectations, and I came away with a more positive impression than I thought I would… the reality exceeded my expectations.” (FG2) “As time has gone by, my expectations have gradually decreased. Each time you go with less enthusiasm.” (FG2)
Relationship with Staff	Treatment by nursing staff	“I have experienced genuine disrespect towards students—negative and invalidating comments.” (FG2) “I have seen some disrespect simply because I was a student… but it was with particular individuals, not with the group in general.” (FG4)
	Inclusion in the team	“There were placements where I felt so well integrated that I would even like to work there.” (FG2)
	Discrimination or rejection	“Are you a student? Then never mind, nothing.” (FG1)
Autonomy and Learning	Level of autonomy	“I find independence contradictory… some nurses are limiting… but others trust you excessively.” (FG2) “The independence they gave me overwhelmed me a lot… I did not even know how to talk to the patient and they were telling me to go and cannulate or draw blood on my own.” (FG3)
	Learning opportunities	“I never had the opportunity to perform the procedure because the following day I was assigned to someone else.” (FG3) “The tutor was very present in everything… the tutorials were real.” (FG4)
	Assessment of competencies	“There is no checklist where someone actually observes how you perform a procedure or assesses your level of competence.” (FG1)
Structure and Organisation	Duration of rotations	“For me, the ideal rotation is three weeks… being two months in the same service becomes excessive.” (FG2)
	Variety of clinical settings	“There are people who never get to rotate through paediatrics… they will never have that experience during their university placements.” (FG4)
	Proposals for improving planning	“Instead of four two-month rotations, I would prefer eight one-month rotations so that we could experience more clinical settings.” (FG2) “I would appreciate being closer, but I would not restrict myself, because I might lose opportunities to experience other services that are farther away.” (FG1)
Assessment of Clinical Placements	Assessment methods	“They are not assessing your clinical practice; they are assessing your ability to write a document.” (FG2)
	Reflective assignments	“We only associate the PAE with a form of assessment… we were not taught how to do a PAE correctly.” (FG1)
	Perceived fairness in assessment	“I was assessed by a tutor whom I had never even met.” (FG3)

FG: focus group; PAE: Proceso de Atención de Enfermería (Standardised Care Plan).

## Data Availability

Given the nature of this research, the data used in this study are confidential and are protected by the research team in accordance with Spanish regulations. They will not be disclosed or shared in order to safeguard the privacy of the participants.

## Supplementary Materials

The following supporting information can be downloaded at https://www.mdpi.com/article/10.3390/nursrep16090337/s1, Supplementary Table S1: Semi-structured interview guide; Supplementary Table S2: Focus group discussion guide.

## Public Involvement Statement

No public involvement in any aspect of this research.

## Guidelines and Standards Statement

This manuscript was drafted in line with the COREQ guidelines for qualitative studies.

## Use of Artificial Intelligence

During the preparation of this manuscript, the authors used ChatGPT (OpenAI, San Francisco, CA, USA; GPT-5.5, accessed August 2026) to improve the language and readability of the text. In addition, the AI-assisted conversational functionality integrated into ATLAS.ti^®^ (version 25.0.1; Scientific Software Development GmbH, Berlin, Germany) was used in both phases of the study to support the qualitative analytical process. AI-generated suggestions were subsequently reviewed against the original anonymised transcripts by the research team, and the final codes, categories, themes, and interpretations were determined by the researchers, as described in Section 2.5. No AI tool independently determined the final analytical framework or interpretation of the study findings. The authors have reviewed and edited all AI-assisted output and take full responsibility for the content of this publication.

## Abbreviations

The following abbreviations are used in this manuscript:

CLE	Clinical learning environment
COREQ	Consolidated Criteria for Reporting Qualitative Research
ECTS	European Credit Transfer and Accumulation System
FG	Focus group
ICU	Intensive care unit
MUs	Meaning Units
OSCE	Objective Structured Clinical Examination
PAE	Proceso de Atención de Enfermería (Standardised Care Plan)
ULPGC	University of Las Palmas de Gran Canaria
