# Supplementary material for: Perceptions of Nursing Undergraduates Regarding Their Clinical Placements: A Qualitative Interpretative Study
Source: Nurs Rep. 2026 Sep 15;16(9):337. doi: 10.3390/nursrep16090337 (PMC13610415; doi:10.3390/nursrep16090337)
Supplement: Supplementary file 1 [file nursrep-16-00337-s001.zip › nursrep-4512856-supplementary.pdf]

Supplementary Materials

Supplementary Table S1. Semi-structured interview guide.

Section	Questions
Personal experience	How would you describe your overall experience of clinical placements so far? What expectations did you have before starting your clinical placements? How have those expectations changed following your experiences?
Relationship with the healthcare team	How would you describe your interactions with the nursing staff during your clinical placements? Can you recall any particularly negative experiences that you would like to share?
Assessment	How are clinical placements assessed in your current academic year? (e.g., fourth-year students complete a Standardised Care Plan (PAE), whereas second-year students do not). Do you think the current assessment methods adequately reflect your learning and clinical performance? How would you describe your experience with your assigned clinical tutor(s)?
Organisation and structure	Do you consider the duration of each placement and the number of clinical rotations to be appropriate? How easy or difficult was it to balance clinical placements with your personal life? Did you receive adequate support whenever difficulties arose?
Choice of clinical placements	Did the location of your clinical placements create any difficulties (e.g., travel distance)? If so, were any solutions offered? Do you think your placements provided sufficient diversity of clinical settings to gain a broad understanding of the nursing profession?
Final reflection	If you could change any aspect of the clinical placement programme, what would you improve? Is there anything else about your clinical placement experience that you would like to add?

PAE: Proceso de Atención de Enfermería (Standardised Care Plan).

Supplementary Table S2. Focus group discussion guide.

Section	Questions
General experience	How would you describe your overall experience of clinical placements? Which aspects of your clinical placements have been the most positive? Why? Have you had any negative experiences during your clinical placements? If so, could you describe them?
Exploration and refinement of Phase 1 findings and alternative perspectives	Are there any aspects of your clinical placement experience that you consider important but that have not yet been discussed? How would you describe the treatment you received from the nursing staff during your clinical placements? Did you feel included as a member of the clinical team? How would you describe the level of autonomy you were given? What are your views on the duration of the rotations and the variety of clinical settings? How do you evaluate the current assessment methods? Do you consider them fair and effective? Do your experiences differ in any way from the issues or perspectives discussed so far?
Comparison with expectations and further exploration	How do your experiences compare with the expectations you had before starting your clinical placements? Are there any experiences, perspectives, or issues that you think have not been adequately represented in the discussion so far?
Identification of problems	What do you consider to be the main challenges or shortcomings of clinical placements? Are there any challenges other than those already discussed? Which of these have had the greatest impact on your learning experience?
Improvement proposals	Based on the issues discussed, what specific improvements would you propose? How could relationships with nursing staff be improved? What changes would increase students' autonomy? How could the duration and organisation of rotations be improved? What changes would make assessment fairer, more meaningful, and more reflective of students' clinical performance? Are there any other changes that you consider important?
